# A Strategic Path Forward for Hospice and Palliative Care: A White Paper on the Potential Future of the Field

**DOI:** 10.1089/pmr.2025.0030

**Published:** 2025-06-04

**Authors:** Ira Byock

**Affiliations:** Dartmouth Geisel School of Medicine, Missoula, Montana, USA.

**Keywords:** clinical standards, competition in health care, hospice staffing, program integrity, program standards, quality measures

## Abstract

The field of hospice and palliative care in the United States is experiencing serious problems and faces an uncertain future. Quality of hospice care is highly variable. Unethical hospice business practices are common in some regions. Palliative care’s integration within American health care has stalled, despite demonstrating that much better care for seriously ill and dying people is both feasible and affordable. Corrective steps have been halting. Urgent work is needed to safeguard seriously ill patients and their families and ensure quality and reliability of hospice and palliative care programs and services. The moment has come for the clinical specialties and corporate community of hospice and palliative care to chart a strategic path forward. Efforts must start with zero tolerance of fraudulent business and clinical practices that harm vulnerable patients. The four components of this strategic approach are (1) publishing clear clinical and programmatic standards, (2) making meaningful data readily available, (3) driving quality-based competition, and (4) embracing the field’s authentic brand of expert care that fosters well-being for patients and their families. Part I of this white paper examines the root causes of the key problems facing the field. Part II presents the rationale and practical considerations for each of the four components of this strategy. This path forward addresses the hard problems the field faces and enables it to realize its dual mission of caring well for ill and dying people and helping society integrate illness, caregiving, dying, and grieving within a continuum of full and healthy living.

## Introduction

A field of medicine I have worked in since its inception, for over four and a half decades, is faltering and is too important to fail. There are signs that both branches of the field of hospice and palliative care have lost direction and face existential challenges. The quality of hospice care in the United States has become unreliable and, with disturbing frequency, unsafe. The industry associated with hospice care is engulfed by a rising tide of unethical practices and avaricious owners. The impressive growth that the medical specialty of hospice and palliative medicine and interdisciplinary palliative care programs experienced during the first two decades of this century has stalled, despite its demonstrated success in high-profile health care systems. Evidence of the discipline’s value is often ignored within corporate suites, hobbling integration within the nation’s mainstream systems, and thereby limiting its ability to meet the needs of seriously ill and dying Americans.

Even as these problems worsen, corrective steps by the field remain halting—held back by a professional culture that avoids conflict, minimizes internal problems, and congratulates itself too readily. This is the moment for this vitally needed clinical specialty and its associated business community to embrace uncomfortable truths and chart a strategic path forward.

There are plenty of members of the field who would disagree vociferously with the premise that American hospice care is in crisis and palliative care is not succeeding. Some colleagues recoil at assertions of serious problems facing the field, responding with variable degrees of denial and anger, bargaining, or resigned depression. In their clinical practices, these professionals are regularly called to give bad news and recognize that only by accepting unwanted situations for what they are, can people craft a better way forward.

In that spirit, I begin this white paper with an in-depth overview of the substantial achievements and the predicaments facing hospice and palliative care in the United States as context for a strategic proposal.

## Part I: Hospice and Palliative Care at Scale—Progress and Problems

From within the field, the strong reactions evoked by such conversations are easy to understand. Those who practice hospice or palliative care know how therapeutically powerful high-functioning teams can be. Clinicians routinely meet patients who are suffering with devastating symptoms of advanced illness, burdened by daily activities of life and the exhausting demands of their care. Many of these patients are also bewildered by dysfunctions and limitations of the health care system and the insurance coverage they rely on. When patients in such situations meet hospice and palliative care teams, it’s as if there was a break in a dark storm. People in misery typically become more comfortable. Sometimes patients say that they finally feel heard and understood,^1^ and therefore, have a new confidence in their ongoing care. Despite knowing that they are incurably ill, patients receiving hospice and palliative care are often restored to a sense of living—sometimes even a sense of well-being.

The clinical prowess of the field’s clinicians and programs has given rise to important accomplishments at scale. The disciplines of hospice and palliative care have proliferated in numbers of programs and patients served. Nearly half of the approximately 1.7 million Medicare recipients who die annually in the United States now receive some amount of hospice care.^2^ Palliative care programs exist in over 80% of hospitals with 50 or more beds.^3^ In addition to its growth, the field has demonstrated its ability to advance American health care’s Quadruple Aim goals of (1) improving quality of care and (2) patient experience, while (3) reducing per capita costs of care and (4) improving satisfaction among professional caregivers.^4–10^

Simply put, hospice and palliative care programs have proven that much better care for people with life-limiting medical conditions is both feasible and affordable. Although lacking the technological sizzle of functional PET (positron emission tomography) scans, genomics, immunotherapies, or transplantation, hospice and palliative care stands as one of the major health care advances of the latter half of the 20th century.

It is no wonder people who work in the field rise to defend it.

## Losing Our Way

Unfortunately, even as it was experiencing success at scale, the field’s innocence was beginning to wane. Fueled by Medicare reimbursements, hospice quickly grew from a social movement in the 1970s to a mission-driven nonprofit community service, and then to an industry. In the first years of the 21st century, anecdotal instances of problems with hospice care gradually became more frequent. By the mid-2010s, accounts of hospice patients being poorly treated or neglected were no longer uncommon. Government oversight agencies, such as the Office of the Inspector General (OIG) of the U.S. Department of Health and Human Services, began reporting alarming numbers of hospice programs with dangerous structural or operational problems. In its 2019 report entitled, “Hospice Deficiencies Pose Risks to Medicare Beneficiaries,” the OIG found that among 4500 hospices, nearly 20% had one or more serious deficiencies and over 300 were poor performers.^11,12^

Exposés by news outlets, including Kaiser Health and TIME magazine,^13–15^ added names and faces within stories of hospices that rarely visited and failed to come to the aid of patients in distress. Anguished families told of being abandoned and feeling that they had failed to prevent their spouses and parents from dying badly.

## Trends in Ownership Coincide with Quality Concerns

The emergence of for-profit hospice programs in the United States is often pointed to as the source of hospice’s problems. It is true that the trends are contemporaneous, yet causation is more complicated.

For-profit business models of hospice programs began in the 1990s. Early on, virtually all were family-owned businesses, often run by individuals who spoke of hospice as a calling. These companies earned reputations for providing excellent care; many prospered and reinvested profits to grow their programs, thereby expanding services to more patients and families. It seemed like a success story.

By the end of the 20th century, a transformation began as more and more owners of these privately held hospices engaged in initial public offerings selling their companies to investors and reaping large profits. These newly publicly traded companies now had legitimate interests in delivering financial gains to their shareholders. In the past decade and a half, another model of for-profit hospice company emerged as private equity firms began acquiring mostly nonprofit, but also previously for-profit, hospices.^16,17^ For some of these investor-owned corporations, hospice companies are merely a category of business holdings. For instance, Chemed, the publicly traded company that owns VITAS, one of the country’s largest for-profit hospice chains, also owns Roto-Rooter, a nationwide plumbing company.^18^

As a result, in the United States today, there are four prominent ownership models of hospice companies: nonprofit corporations owned by a fiduciary board of directors, private for-profit corporations, wholly owned by an individual or private group, publicly traded, shareholder owned for-profit corporations, and for-profit corporations owned by private equity firms that are not publicly traded.

Quality problems are hardly restricted to hospices owned by for-profit companies. But there is evidence for important associations between hospice ownership and quality of care.^19–21^ Compared with nonprofit programs, on average for-profit hospices employ fewer skilled clinical staff with less training and provide a narrower range of patient and family services.^19^ For-profit programs tend to enroll patients with lower skilled needs, and therefore, less daily costs, for longer periods of time.^22,23^ Patients served by for-profit hospices are more likely to be discharged from hospice care prior to death or experience burdensome transitions of care, including emergency department care and hospitalizations.^24–26^ For-profit programs also generate more complaints and have more serious deficiencies than not-for-profit hospices.^11,27^

A recent preliminary review by the Oregon Health Authority of a proposed joint venture between Providence, the state’s largest provider of home health and hospice and Compassus, a for-profit company co-owned by a publicly traded and a private equity firm, plainly states the situation: “To the extent, providing better care to community members conflicts with profit objectives, for-profit owners would be expected to prioritize the latter. This may drive various cost-cutting and revenue-maximizing strategies that may affect the range of services offered, patient care practices, admissions and discharge policies, locations of health care facilities, employee compensation and staffing, etc.”^28^

Most comparative studies have investigated differences between nonprofit and for-profit hospice programs, without distinguishing the three types of for-profit hospice corporate ownership. However, a recent study found associations of caregiver-reported quality ratings with hospice ownership that extended to subcategories of for-profits. Based on the Consumer Assessment of Healthcare Providers and Systems (CAHPS) Hospice Survey, nonprofits performed better than privately owned for-profits, which performed better than private equity or publicly traded hospice programs on 7 of 8 summary measures, including willingness to recommend the program, getting timely care, rating of the hospice, getting help for symptoms, hospice team communications, treating family member with respect, and getting emotional and religious support.^20,29^

Since over 70% of American hospice programs are owned by some type of for-profit company, it is obvious that if hospice in America is to succeed, for-profit hospices must be able to succeed. We can be confident that this objective is achievable because there are plenty of privately owned and investor-owned hospices companies today that operate well-managed, well-staffed, and well-resourced programs while remaining financially healthy.

Vigilance is required. The Medicare and Medicaid *per diem* form of hospice payment invites monetary opportunism that puts the quality of services and integrity of the field at risk.^16^ A stark example is the variants of privately owned hospices that have recently proliferated in areas pf Arizona, California, Nevada, and Texas. By reasonable expectations of staffing, operations, and performance, many of these programs are hospices in name only.

In March 2022, the California State Auditor noted that “Since 2010 California has experienced an explosive growth in hospice agencies that does not appear to correlate with the need for hospice services.” The situation in California’s southern counties reached extreme proportions. Los Angeles County in 2010 had 109 licensed hospice programs; by 2021 that figure had mushroomed to 1841 programs.^30^ In some instances, multiple hospice programs listed the same address and owner. The OIG and investigative journalists continue to expose hospice companies that aggressively recruit patients who are not terminally ill, an illegal practice that, under Medicare rules, deprives chronically ill people of Medicare coverage for disease treatments that might help them live longer. Physicians employed by or contracted with these programs may have little or no direct involvement in patient care.^31^ Such physicians may be deliberately complicit in improperly certifying patients for the Medicare hospice benefit or, merely disengaged, offer their signatures as a perfunctory service to the hospice programs.

In response to government reports and journalistic exposés, the Centers for Medicare and Medicaid Services paused new certifications for hospice programs in hotbed regions of Southwestern states and launched a Hospice Special Focus Program of heightened scrutiny of poorly performing hospices.^32^ Concurrently, the HHS OIG pursued legal actions against a handful of individuals and companies suspected of fraud.^33^ Taken as a whole, these welcome steps by government have had some, but limited, effect. Few programs have been penalized or closed. The Special Focus Program was paused in February 2025 and later canceled under the new federal administration.^34^ Hundreds of hospice programs across the country continue to use deceitful practices to aggressively enroll patients and then fail to skillfully provide them with the care and services they need. Many more programs are understaffed and nurses are provided inadequate training and deprived of sufficient physician support—conditions that render hospice clinicians and teams unprepared to care well for dying patients and their families.

These are not exaggerations. Over half of all hospice programs in the United States provide neither general inpatient nor continuous home hospice care, the two intensive levels of hospice care that are essential for aiding patients and families in unrelieved distress. This deficit persists, even though the capacity to deliver both services is the regulatory requirements of Medicare’s conditions of participation (CoPs).^35^

In March 2024, the Medicare Payment Advisory Commission reported that only 10% of hospices earned a 5-star quality rating and 39% earned 4 stars, leaving half of all U.S. hospices earning 3 stars (36%) or less. How many clinicians would refer patients to—or want a family member cared for by—a hospice that could not earn at least 4 stars?^36^

I have focused here on problems related to program integrity and quality of hospice services. Before turning attention to the palliative care branch of the field, it is important to acknowledge additional, long-standing problems. Hospices in the United States wrestle with persistent patterns of late patient referrals and demographically uneven access to hospice services. Access to—or acceptance of—hospice care is diminished for people of color, minority ethnicities, and those who don’t speak English, as well as those who live in rural communities. Overall, half of patients admitted to hospice care receive services for 18 days or less—a figure that has not budged in decades—and a quarter of enrolled patients receive 5 days or less of hospice care.^2^

## Palliative Care Rapid Development and Growing Pains

Palliative care’s growing pains are less dramatic, but, seen from a population health perspective, nevertheless worrisome.

Since the mid-1990s, palliative care’s evidence base has expanded in breadth and depth.^37^ Refined clinical assessment tools and research methods enabled comparative studies of treatments for physical distress, anxiety, depression, and delirium that have improved care and experience of people living with serious illness. Meanwhile, studies showing that hospital-based palliative care is associated with significantly lower total costs of patient care helped to drive significant growth in the number of palliative care programs, particularly within academic centers and larger community hospitals.^7,8^ In 2002, the Veterans Health Administration began offering hospice and palliative care services to seriously ill patients throughout its national system of hospitals and home-based primary care programs, without a requirement to forego disease treatments.^38^

At present, nearly 84% of hospitals with 50 beds or more report having a palliative care program, although percentages vary by geography, hospital size, and ownership.^3^ The Center to Advance Palliative Care (CAPC) publishes a Serious Illness Scorecard of state-by-state variations in palliative care capacity based on availability of specialty-trained professionals, basic palliative care education for all clinicians, payment for palliative care services, support for patients’ functional needs and their caregivers’ needs, and support for palliative care awareness.^3^ Palliative care programs vary widely in clinical team composition by disciplines and numbers of clinicians, specific patient services they provide, and days and hours of availability.^39,40^ This degree of variation means that within an average moderate-to-large American city, there is likely to be at least one medical center with a well-staffed, high-functioning palliative care program, as well as other hospitals without any palliative care program, or ones that are so thinly staffed and functionally limited as to represent palliative care in name only.

As a young medical subspecialty, hospice and palliative medicine is not growing rapidly enough to meet either the current or future needs of the country’s population of aging and chronically ill people. At present, there are an estimated 7000 hospice and palliative medicine physicians in practice.^41^ Nearly 200 hospice and palliative medicine (HPM) fellowship programs graduate about 400 new HPM specialists each year.^42^ While this represents significant progress, an estimated 18,000 additional specialty palliative medicine physicians will be needed in the next decade.^41^

This predicament was summarized succinctly by Dr. Eduardo Bruera in a September 2024 editorial in JAMA, “For those of us who have worked in palliative care for decades, the critical unanswered question is this: With clear and consistent evidence of improved clinical outcomes, quality metrics, and financial outcomes with palliative care and additional evidence to support accessible and scalable interventions, what more will it take for executives, insurers, and regulators to finally support palliative care programs?”^43^

The serious problems confronting hospice and palliative care in the United States are not unique to the field, but instead, are manifestations of systemic dysfunctions within the nation’s health care systems.^44^ Greed is the systemic infection that affects every medical specialty and frustrates progress toward each of the Quadruple Aims.^45,46^ Talk with any practicing physician and you will hear myriad ways in which the pursuit of profits—usually under the guise of efficiency and productivity—adversely affects patient care and their own professional satisfaction. Joy at work, the fourth of the Quadruple Aims, is a receding fantasy, replaced by hopes of at least ameliorating work-related depression and moral distress among clinicians.^3^ These systemic dysfunctions persist because, from the perspective of owners and investors of corporate health care organizations, avarice works. Unfortunately, it works to the detriment of patients, their families, and professional caregivers.

## Part II: A Strategic Path for Hospice and Palliative Care

We need not succumb to depression. This understanding of the history and underlying sources and forces of these problems suggests a strategic path forward. The clinical disciplines and associated industries of hospice and palliative care do not have to solve American health care’s maladies to safeguard and ensure that seriously ill people and their families receive competent, reliable care.

In the remainder of this essay, I discuss the rationale and application of a strategy the field could adopt to restore its reputational integrity, ensure quality and reliability of its services, and realize its transformational potential. The four components of this strategy are (1) publishing clinical and programmatic standards, (2) making meaningful data readily available, (3) fostering quality-based competition, and (4) embracing and promoting an authentic brand.

This strategy is straightforward but not simple. It will require a fair degree of agreement among the field’s clinicians and business and trade associations, as well as relevant oversight agencies, academicians, technical consultants, and patient advocacy groups. Anything approaching consensus will require these diverse stakeholders to prioritize what is best for seriously ill patients and their families. Published standards and corresponding measures will represent—*must represent*—innumerable endless refinements, revisions, and iterations. The sooner we start, the better (Table 1).

**Table 1. Summary of the Four Components of a Strategic Path Forward:** 

Strategic component	Rationale	Practical considerations
Clinical and programmatic standards	Standards provide the basis for meaningful evaluation of quality and accountability in hospice and palliative care. Without operational specificity—including minimum staffing ratios, training hours, and response times—existing published guidelines fall short of what this strategy requires.	Broad stakeholder involvement is essential, including those with divergent financial interests. Timelines for development and review of standards should be clearly defined and adhered to. Published standards must include scheduled updates to maintain relevance and impact.
Making meaningful data readily available	Data and measurement allow assessment of performance against published standards. Accessible, reliable, and user-friendly public-facing data enable patients, referring providers, and payers to make informed choices.	Data must be trustworthy and resistant to manipulation. Imperfect data can still guide choices; data, analyses, and rating scales should evolve over time. Comparative data must be easy to navigate and clearly communicated to nonexperts.
Driving competition based on quality	Currently, financial interests dominate competition in hospice and palliative care. Reorienting market success to align with measured quality of services and patient–family experience is essential. For-profit and nonprofit providers alike must compete by delivering demonstrably excellent care.	This transformation to quality-based competition requires coordinated efforts from professional associations, regulators, payers, and patient advocacy groups. Success hinges on active promotion and reliance on transparent quality data across health care stakeholders.
Embracing and promoting our authentic brand	Public perceptions are of prime importance to quality-based competition. The field is distinguished by intentionally fostering well-being for people it serves. In embracing this distinctive identity, hospice and palliative care can establish itself as an essential service for people with serious illness.	Rebranding publicly reintroduces a product or service. A refreshed brand that is honest and uplifting could move hospice and palliative care from “nice to have” to “must have” specialty services. Promoting well-being can also redefine public and system-level expectations of quality care.

### Zero tolerance

Zero tolerance is not actually a component of this strategic plan; it is a prerequisite. The field must adopt and adhere to an explicit zero tolerance stance toward fraud and abuse by hospice and palliative care programs. Boundaries of collegiality stop at criminality and patient harm. Competition within health care must occur within ethical and legal constraints.

Although it is the job of government agencies to enforce laws and regulations, the professional field and related industry of hospice and palliative care must help to expose acts that harm vulnerable patients. When clinicians see something suspicious—such as using deception to enroll patients who are ineligible for hospice care or abandoning patients when their hospice care becomes too expensive—they must say something. The field can educate itself to recognize telltale signs of fraud, abuse, and negligence and set up “hot lines” for reporting suspected cases to the appropriate oversight agencies.

#### Clinical and programmatic standards

At the core of this strategy are explicit guidelines for the structure and processes of hospice and palliative care practices and programs. As difficult as it will be to reach an agreement among diverse stakeholders who have conflicting financial interests and, let’s be honest, varying commitments to quality, everything else depends on accomplishing this step well.

Standards and best practices for a health-related field and industry provide the framework for meaningful measurement and comparative evaluation and undergird effective accountability. They are necessary to build a level playing field for competitive markets based on quality. Conversely, in the absence of clear, measurable standards, who is to say that any health care program or services are substandard? That is the unfortunate situation we have today.

Responsibility for establishing minimum specifications and best practices for safe and effective care squarely belongs with each medical specialty and health care service line, acting through their professional and industry associations. Unfortunately, the field of hospice and palliative care has consistently shirked this responsibility. Against the backdrop of prevailing problems in American health care, oversight agencies, payers, and the public should be wary of any medical specialty or health care service association that declines to publish clear clinical and programmatic standards.

An existing set of Clinical Practice Guidelines for Quality Palliative Care published by the National Consensus Project is broadly categorical and qualitative, even in matters related to the staff composition of interdisciplinary teams and response times for screening, assessment, and treating of symptoms, that would seem to call for numerical ranges.^47^ Nevertheless, these guidelines provided a framework that enabled the Measuring What Matters project, a collaboration of the American Academy of Hospice and Palliative Medicine and the Hospice and Palliative Nurses Association, to recommend validated quality indicators for key aspects of hospice and palliative care.^48^ These efforts set the stage for explicit standards that can be used in objectively evaluating and comparing programs.

While serving as a member or advisor to various quality committees during the 1990s through 2010s, I repeatedly witnessed executives and boards of the field’s national associations turn down calls to publish unambiguous programmatic standards. This is an opportunity to leadership of these associations to consider how often current Centers for Medicare and Medicaid Services (CMS) hospice accountability surveys and audits miss their mark, adding to a hospice program’s administrative burden and costs of delivering care without improving quality an iota. In a similar vein, lacking crisp parameters to apply in identifying bad actors, Medicare’s efforts, such as the now-paused Hospice Special Focus Program, risk a “ready, fire, aim” approach that could damage much-needed remediation efforts.^49^

##### Hospice care standards and best practices

In the hospice discipline of the field, a starting place is the Medicare Hospice Benefit CoPs. The CoPs describe rudimentary nuts and bolts of clinical teams and programs, including that programs maintain a complete interdisciplinary team. Importantly, the CoPs stipulate that programs must be able to provide all four levels of hospice care and offer bereavement services for families.^30^

If they committed to the process, seasoned hospice and palliative care clinicians and administrators could readily draft a set of specific standards and guidelines that detail elements of program structure and functions necessary for effective operations and quality outcomes. These would specify minimum criteria for interdisciplinary team staffing, along with optimal discipline-specific caseloads and patient–staff ratios. They would include discipline-specific minimum and recommended frequencies and durations of patient visits. Such standards could incorporate adjustments for challenging practice settings (i.e., long drive times) and patient acuities. Programmatic standards should also describe the processes and corresponding resources required to respond to urgent patient or family needs and symptom crises.

##### Hospice nurse staffing

Numerical parameters for staffing and caseloads for nurses and other hospice clinical staff will likely be the most contentious details in this step of this strategy. Since personnel is the most expensive line item in any hospice program’s budget, owners and boards of hospice companies often see higher caregiver caseloads as a way of containing costs, or in management speak, improving efficiency and productivity. Some managers assert that innovations in clinical charting, telehealth, and team-based care may enable hospice nurses to shoulder caseloads of 15–20 patients.

Across the hospice industry at present, it is not uncommon for a hospice nurse case manager to be responsible for 16–18 patients at a time and caseloads of 24 patients are not rare.^50,51^

These numbers are far above the clinical workloads of hospice teams I worked within during the 1980s and 1990s. Wondering whether my clinical perspective was antiquated, I consulted Multi-View Inc., an operational benchmarking company that serves the hospice industry. Multi-View’s online Caseload Expectations reference table lists 12 hospice patients per nurse as acceptable and 14 per nurse as excellent from an efficiency perspective.^52^ I also interviewed Donna Morgan, a nurse executive with 28 years of hospice experience, and Susan Cox, who has 20 years of hospice experience and is Chief Nursing Officer for a program with an average census of 260 hospice patients.^53^ Each emphasized the importance of numerical staffing standards for care quality and staff well-being. Without referencing the Multi-View benchmarks, they also identified 12–14 patients per hospice nurse case manager as their programs’ targets. They added, regretfully, that they were not meeting those targets. Recruiting challenges and the national nursing shortage, which worsened during the COVID-19 pandemic, were forcing them to have nurses carry caseloads of 17–20 patients. Each said that their program had a formal hiring and retention plan in place to meet staffing goals.

Staffing guidelines must extend to numerical minimums and best practices for the training of clinicians and aide-level caregivers. Morgan told me that she is alarmed that some programs are requiring just six weeks of on-the-job training for nurses without previous hospice experience. “I can’t imagine a nurse with no hospice experience being prepared to go out there and carry your case load in six weeks,” Morgan said, adding, “I don’t think they have enough ability to be carrying a full case load of even 12 to 14 until four to six months …” Cox said that it takes at least 12 weeks of on-job training for a hospice nurse to manage a caseload of 8–10 patients and weeks to months more to effectively carry a full caseload of 12–14 patients.

Hospice nurses leaders have told me that, at present, little or no orientation is not rare. A new hospice nurse expressed her plight on a popular hospice blog, “I oriented in the field for two weeks with the DON [director of nursing]. After that I got my 18-patient caseload. Even with help from my team I am drowning and miserable.”^54^

##### Roles and responsibilities of hospice physicians

The roles and participation of doctors within many, but not all, hospice programs have steadily receded over the years. While nurses have always been the core of clinical teams, actively involved, skilled physicians are integral to good hospice care. In fact, ensuring that dying patients have access to knowledgeable doctors was a key impetus for developing hospice as a specialty service. Since dying patients are, by definition, among the sickest patients in our health care systems, they deserve the attention of doctors. Currently, in many hospice programs, insufficient physician involvement leads to delays and failed responses to urgent patient needs, complaints from patients and families about being unable to see a doctor, and hospice nurses feeling inadequately supported.

Standards and guidelines should enumerate minimums and best practices for both hospice physician administrative and clinical responsibilities.^55^

It is important to acknowledge that advanced practice providers (APP) are needed to expand the hospice and palliative care workforce. Nurse practitioners (NP) and physician assistants (PA) can extend the reach of hospice physicians, and their numbers can be factored into staffing guidelines.

At present, APPs may still encounter barriers, such as limited scope of practice and prescriptive authority based in statute or policy in some states.^56,57^ Many would benefit from additional specialty training in hospice and palliative medicine. In a national survey of advance practice registered nurses, 39.2% of respondents felt that their graduate education adequately prepared them to practice in the specialty and 61.5% indicated that they had no palliative care content in their graduate courses.^57^ An expert panel suggested that a minimum of 150 practicum hours under oversight by a palliative care specialist are needed.^58^ Programmatic standards encompassing core content and minimum hours of structured onboarding and clinical mentorship for advance practice providers are essential for maximizing their clinical contributions to HPM interdisciplinary teams.

Even when able to practice at the top of their licensure, involvement of these APPs is not a justification for replacing doctors. On the contrary, in my experience, the highest functioning clinical hospice teams include NPs or PAs who practice in close collaboration with hospice physicians.^37^

##### Palliative care standards and best practices

The palliative care division of the field has developed without certification requirements or substantive Medicare regulations. Two private accrediting organizations offer elective certifications for palliative care programs. The Joint Commission and the Community Health Accreditation Partner evaluate and offer certification to hospital-based palliative care programs, as well as programs that are based in hospice and home health agencies, assisted-living, or skilled nursing facilities.^59,60^ Because these organizations’ criteria are proprietary and intended for high-functioning palliative care programs that desire formal recognition, they do not fill the need for minimum structural and functional standards across the field.

As with the hospice division of the field, palliative care program standards must include explicit minimum and best practice staffing levels for each of the clinical disciplines that comprise a palliative care team, operational days and hours, and roles and responsibilities of each categorical member of the team. Guidelines should specify routine use of established symptom assessment tools and require provisions for responding to after-hour patient needs.

Most basically, the field must publish criteria for calling something a palliative care program. Professionals in our field commonly experience real-world effects of the wide variation in palliative care programs when trying to gain access to palliative care for a relative, friend, or colleague in another state or unfamiliar health system. We may be told that the program does not accept self-referrals from patients or families. Or that the program can only accept referrals from their own health system’s providers. Or that the program only sees outpatients as part of a multidisciplinary cancer clinic or heart failure program or does not have an outpatient practice at all.

While preparing this white paper, I tried to help a friend access palliative care for her husband, who was being treated for recurrent head and neck cancer at a National Cancer Institute (NCI) Comprehensive Cancer Center on the east coast. He was experiencing daily pain, dry mouth, along with mood changes and anxiety about his disease and future. His surgical and oncologic teams had never mentioned palliative care.

On the cancer center’s website, there was only a boilerplate description of palliative care, without any contact information or list of the program’s services or clinicians. I called the cancer center’s main number, but the receptionist was unfamiliar with the term palliative care and couldn’t find an office number for such a program. The next day, I tried again, and another receptionist did find a number for palliative care. I called that number twice during business hours; it was answered by voice mail. I left my name and number but never received a call back.

As it happens, there is another NCI Comprehensive Cancer Center within an hour’s drive from my friend’s home. That center’s website includes an extensive page for palliative care with a prominent phone number for questions and appointments. There are thumbnail photos for a full complement of clinician team members, including the program’s 20 faculty physicians and 7 advanced practice nurses. I passed this information along to my friend.

Lacking a clear definition and operational criteria for palliative care, both of these cancer centers can assert that they have a palliative care program.^39^ Surveys that rely on self-reporting cannot distinguish real from ersatz palliative care programs. Unless and until one tries to obtain services for an individual patient, key programmatic details that impact access to palliative care go unseen (Table 2). Let patients and families beware.

**Table 2.** Program Factors Impacting Access to Palliative Care

Factors impacting access to palliative care
Ease of contacting palliative care program
Medical center’s website has a page for palliative care that includes: ◦ Phone number and contact information ◦ Office hours for the program ◦ After hours contact instructions Medical center receptionist or phone operator can transfer caller to a PC office
Access friendly referral procedures and scope of services
Maintains an outpatient clinic Accepts self-referral of patients and families for outpatient care Clinician available by phone for after-hour questions Physician or APP available on weekends to consult on hospitalized patients

The strategy I am proposing anticipates a future in which the presence of a functional palliative care program is an important factor for a patient who must choose between available cancer centers, a doctor who must decide where to refer newly diagnosed patients, and a Medicare Advantage plan that is building a preferred provider network.

Steadfast leadership within the field will be required to publish standards with sufficient specificity required for oversight activities and making choices in competitive health care markets. Commensurate with the urgency of correcting deficiencies and meeting the needs of seriously ill patients and families, the field would be wise to set an aggressive timeline for accomplishing this goal for both hospice and palliative care clinical divisions of the field.

#### Making meaningful data readily available

Standards and best practices paint a landscape and draw architectural blueprints for a health care field as it is envisioned and intended to be. Measures, data, and analyses reveal the topography of a field as it is. Measured gaps between current operations and outcomes and standards reveal the faults and focus corrective actions and continuous quality improvement.

Extending the topographic analogy, data shine a light that shows a specialty’s terrain in three-dimension by illuminating access, quality, and costs in bar graphs, pie charts, tables, and dynamic dashboards. Zooming in we view granular information of caseloads, response times, annual staff turnover rates, and programmatic outcomes. Zooming out, comparative data pertinent to oversight and customer choice are visible through quality indices, satisfaction scores, and rating scales.

Relevant data must be as reliable as possible—accurate and difficult for programs or companies to “game”—readily available and easy to understand. Rating scales and corresponding designation criteria for stars, precious metals, or colored ribbons should be works in continual refinement. Perfection must not be an excuse to delay this strategy.

Public facing sites can present comparative data on both hospice and palliative care programs in ways that are easy to understand. Sites should include basic descriptive information about ownership, each program’s years in operation, size, total number of full-time-equivalent staff by discipline, and range of specific services provided. Reflecting the field’s most current programmatic standards, information can include periodicity of IDT meetings, frequency of patient visits, and physician involvement in specific tasks of care planning, clinical team support, and direct patient care.

Outcome data for accountable care organizations, health insurance plans, Medicare Advantage plans, and population health programs can include average and median days that patients receive hospice care and palliative care services. These data complement comparative data on the percentage of days that patients spend in an acute care hospital and ICU during the last 30 days of life, and the location of their death. For hospice programs, outcomes should include the annual percent of general inpatient and continuous home care days of service delivered.

Patient-level clinical outcomes can include aggregate changes from baseline over time in patient-reported scores for pain, dyspnea, depression, anxiety, demoralization, spiritual distress or well-being, and global quality of life. Patient-reported information on feeling heard and understood is increasingly recognized as a core reflection of patient experience.^1^

Surveys of family members after a patient dies can be an important source of information on quality of care and patient experience. Medicare’s CAHPS Hospice Survey assesses the experiences of patients who died while receiving hospice care through a survey of their informal primary caregivers. The CAHPS transforms aggregated subjective experiences into numerical 1–5 scores and awards star ratings based on “top box” scores. A derivative “net promoter” score, which is commonly referenced by health care quality experts, is the top box ratings on the item, “would you recommend this hospice” of the CAHPS survey.^20,29^

Medicare also maintains a separate claims-based Hospice Care Index (HCI) set of star ratings that assess 10 processes of care, skilled visits during the last 7 days of life, any visits, continuous home care and (general in-patient) GIP care during the last 3 days of life, frequency of early and late live discharges, and gaps in skilled nursing visits.

Both CAHPS and HCI star ratings are important existing resources that demonstrate the potential for public-facing quality information to influence choice and referral patterns. They deserve to be more widely promulgated, despite being limited by methodologies that lack of case mix adjustments and sampling that omits many rural or otherwise low volume hospice programs. (An exclusion that is exploited by some unscrupulous hospice programs to avoid surveillance.)^61^

The main current limitation is that having two rating scales based on different, albeit complementary, criteria is confusing. The National Hospice Locator, a project of Hospice Analytics, is an attempt to harmonize public-facing data from the same experience surveys and claims data along with salient corporate information within a format that is easier for referring providers, discharge planners, patients, or families to use.^62^

Future rating scales should include key measures related to program staffing. The annual percent turnover of nurses and aide-level personnel has been applied in comparing skilled nursing facilities and would be of value here.

#### Driving competition based on quality

Competition is the engine of this strategy, using the energy of capitalism to drive hospice and palliative care toward high quality. Imagine a near future in which the only way for a hospice program to succeed was by consistently providing *measurably* high quality of care to patients and families. This future, easy-to-understand, online data about performance of every hospice program are immediately available for doctors and hospital discharge planners to use in making referrals and for patients and families to use in choosing among local hospice programs for their care. Imagine insurance companies and health plans using such data in deciding which hospice providers to include in their networks, knowing that they’d face blistering criticism if they excluded the best programs. In this future state, reliably high quality is the principal factor determining a hospice or palliative care program’s financial success.

In the present state, competition within health care markets and among hospice and palliative care companies and programs is complex and murky. Contracts and referral pathways turn on name recognition and intercorporate relationships. A mixture of advertising and direct marketing also influence patient referrals. A prevalent erroneous belief that CMS regulations forbid hospitals and discharge planners from recommending specific hospice programs to patients and families based on quality has inhibited fair competition in hospice markets.^63^ Financial priorities are the constants that run through competition with success measured in profits, stock values, and bond ratings. Corporate deals are made and volume discounts offered. To a bond rater or stock trader, staffing levels are overhead, patient services are necessary expenses, and quality ratings are a public relations matter. In the largest corporations, occasional regulatory fines are accepted as a cost of doing business. The lived experiences of sick patients and the satisfaction of skilled professionals are filtered out by lenses of competition that is based on financial margins.

Speaking with executives of publicly traded and private equity health care companies at conferences and listening to their comments on conference panels can leave the impression that hospice and palliative care programs are viewed as bit players in American health care, pieces on the board to be moved or traded for market share and financial gains. Often, it is only when they or a member of their family becomes gravely ill, do senior corporate executives or financial overseers realize that hospice and palliative care are critical to a person’s quality of life during advanced illness; the difference between life worth living and dying badly.

Americans can assume that the country’s health care system will remain dominated by large corporations for the foreseeable future. This is all the more reason for quality to become the currency of competition.

Accomplishing such a shift is analogous to moving mountains, in this case, the corporations and conglomerates that dominate America’s health care landscape. Current competition, which is based on solely financial considerations, has proven to be fertile ground for mergers and acquisitions, corporate agreements, and business practices. This *status quo* is unlikely to give way to an incremental picks-and-shovels approach of regulatory reforms. The operating environment and assumptions on which health care businesses are built must shift to enable quality of care and patient experiences data to become key determinants of the financial success and viability of clinical programs and companies.

In a future state in which the competitive playing field of health care is level, the ownership and tax status of a hospice program becomes a lot less relevant. This four-point strategy is designed to do just that. At present, however, ownership matters. In the short-term, at least, public-facing transparency is needed about both the category of ownership and the specific companies and parent corporations that own each program.

To successfully steer the hospice and palliative care business community toward safe, reliable, and effective care, quality data relevant to programs’ structure, operations, and outcomes must not just be readily available but also be actively promoted to referring providers and patients and families. The success of this strategy will turn on the extent to which the major stakeholders in the safety, quality, and value of health care—such as Medicare and Medicaid, health care specialty associations, nongovernmental quality organizations, public health organizations, and patient advocacy groups—endorse and rely on relevant quality measures, reports, indices, and ratings that were developed by the field.

Consider the impact that might occur if a coalition of the field’s national organizations, such as the National Alliance for Care at Home, the American Academy of Hospice and Palliative Medicine, and the Hospice and Palliative Care Nurses Association, were to jointly publish a patient-facing handout of “Questions to Ask Before You Sign—Help in Choosing a Hospice Program,” that included questions such as:

- What company owns the hospice program?
- How many patients is our hospice nurse caring for?
- Will a physician visit us at home?
- Who will visit us if our hospice nurse is away or not available?
- Who do we call with urgent questions or problems?
- What happens if the medicines we have aren’t working?
- What is our “crisis management” plan?
- Is there a dedicated hospice facility available if needed?
- Will our family receive bereavement support?

This strategic approach encourages all hospice programs—whether they are nonprofits, privately held for-profits, shareholder owned, or private equity owned—to succeed by putting patients and families first—and proving it—through measured patient experiences, quality outcomes, clinician satisfaction, employee retention, and, of course, star ratings.

#### Embracing and promoting our authentic brand

While the engine of this strategic approach is competition based on quality, people’s perceived needs and desires are fuel for the choices they make. Public perceptions are of prime importance to competitive success, which is why brands matter. A product’s or service’s brand expresses how it wishes to be known by the public—and potential customers. Brands strive to convey positive meanings and emotions.

Since its inception, the field of hospice and palliative care has been unable to coalesce on a brand. The field remains conflicted about its own names, reflecting an anxious sense that the public and potential patients are afraid of what we do and represent. Public relations and marketing practices that are vague or use euphemisms inadvertently perpetuate this situation by leaving an impression that hospice and palliative care programs have something they are reticent to talk about.

Despite occasional public relations campaigns asserting that “Hospice is about living,” the word hospice is undeniably associated with dying and death. Among the public and within the business community, death remains a hard sell. Within health care, death avoidance underlies the reluctance to refer patients to hospice or palliative care, and patients’ and families’ resistance to meeting hospice or palliative care clinicians. This psychological barrier delays hospice and palliative services to patients and families who are later pleasantly surprised, saying, “I wish we had known about you earlier.”

This psychological discomfort is not limited to the general public, patients, and referring physicians; rather, it permeates the field itself. Too many hospice personnel are uneasy about having the word “hospice” on their name tags. Some clinicians in the field wonder if they should avoid the saying “hospice” in introducing themselves to patients and families. A senior physician told me that he has urged the Academy of Hospice and Palliative Medicine to drop “hospice” from its name, explaining that “Palliative medicine is what we practice; hospice is where some of us work.” Based on marketing research, the CAPC urges palliative care providers to avoid mentioning hospice and palliative care in the same breath to avoid the associations that the word “hospice” has with terminal illness and dying.^64^

There are also leaders in the field who worry that palliative care has been tarred with the same death and dying brush. The perception that the term “palliative care” also scares patients has given rise to debates about whether the field should rename its programs.^65^ In fact, a study at M.D. Anderson Cancer Center, found that adding supportive care to the palliative care program’s name, resulted in a 41% increase in referrals.^66^

Without engaging in the pros and cons of the question, we can appreciate the irony of worrying about the term palliative care in a medical facility that proudly calls itself a cancer center. Few words evoke more fear in patients than “cancer.” Admittedly, two exceptions are the words “death” and “dying.” That is the reason that CAPC also advises clinicians and programs to avoid saying “dying,” “terminal illness,” or “end-of-life care” when talking about palliative care.^64^

These discussions are reasonable. Yet, it seems that the field has painted itself into a cultural corner. Hospice and palliative care professionals are rightly proud of the work they do but afraid their disciplines’ names will strike fear in others. To wit, when the National Association for Home Care and Hospice and the National Hospice and Palliative Care Organization recently merged, the National Alliance for Care at Home was formed. Gone from the new organization’s name and mission and vision statements were any mention of hospice or palliative care.^67^

Let’s look for a way out of this cultural dead end. The problem is that it’s difficult to honestly explain palliative care without mentioning that the clinicians and teams care for patients with serious, potentially life-limiting conditions. Since hospice programs admit people who are formally determined to have a “… life expectancy is 6 months or less if the illness runs its normal course,” avoiding terms like “dying” and “end of life” requires semantic contortion.^68^

This situation is primarily an important clinical communication challenge and, secondarily, a marketing challenge. However, solving this conundrum would have far-reaching, salutary social and cultural implications. It is important to remember that the field was born with a dual clinical and cultural mission. At the time, John Hinton’s *Dying* (1967), and Elisabeth Kübler-Ross’s *On Death and Dying* (1969) awakened the medical community and public to endemic clinical deficiencies in care dying people received.^69,70^ Socially and culturally, Ernest Becker’s *The Denial of Death* had similar effects.^71^ Hospice and palliative care arose to care well for the most gravely ill people in our society, *and in the process*, to help integrate illness, dying, caregiving, and grieving within the social–cultural continuum of human life. In practice, these objectives are inextricably linked—progress in one generates progress in the other—and failure of one begets failure of the other.

##### Hospice and palliative care’s authentic brand

I suggest that the field of hospice and palliative care is distinguished by its practice of intentionally fostering well-being in the people it serves. This focus is so embedded in the assumptions, attitudes, and practices of the field’s clinicians and teams that it is largely unrecognized. Simply put, health care that fosters well-being through the end of life is hospice and palliative care’s brand.

Clinicians from a variety of health care specialties can competently care for seriously ill and dying people. Skillful symptom management, clear communication, and shared decision-making are sometimes referred to as primary palliative care; regardless of the clinician’s specialty, these skills constitute good medical practice. Hospice and palliative care are set apart by their understanding that illness and dying are profound personal experiences for patients and families, filled with risks and potential suffering, as well as potential opportunities. Clinicians in this field are not just symptomatologists. They approach patients and their families as whole persons who have important personal relationships, as well as inner lives. Clinicians and teams approach illness, caregiving, dying, and grieving not as unwanted but as normal parts of peoples’ lives that are impacted—but not entirely defined—by their diagnoses and medical needs.

Clinicians in the field recognize that receiving a bad diagnosis is sometimes a wakeup call, prompting a person to take stock of what matters most for them. Usual personal priorities may fall away and the “things that need to get done” on any given day tend to change. Some people swiftly retire, turning over responsibilities and special projects to others, thereby becoming free to invest time and energy in long-held desires.

Perhaps because there are no billable codes for the tasks involved in fostering well-being, that term is rarely used. Nevertheless, skilled hospice and palliative care clinicians and teams purposefully help people do what they need to do to feel complete and at peace within personal realms of their lives. This is not a platitude; it is an explanation and description of what clinicians and teams plan for and practice in caring for ill people and their families.

##### Examples abound

A clinical team may help a patient attend a child’s or grandchild’s graduation, piano recital, or school play, Bar Mitzvah, or quinceañera. I was part of a palliative care team that planned and prepared for weeks to enable a man, who had undergone pelvic exenteration surgery and required ostomies and fistula drainage bags, and was in constant pain, to walk his daughter down the aisle at her wedding. Twice during the same year, our team organized in-hospital weddings for a dying patient and fiancé. Support of this sort is unexceptional, indeed common, in high-functioning hospice and palliative care programs across the country. It is also common for clinicians to counsel patients, who desire to do so, to reach out to estranged relatives or friends to mend fractured relationships. They may help a person to craft a letter or prepare for a phone call. Some patients appreciate help in planning their own “going away” parties. Often families value support in holding a vigil for a person who is actively dying.

To those who comprise the field, all of this seems natural and like nothing special, but it is the distinguishing feature of hospice and palliative care. If there were billing codes for honoring and celebrating people who are ill, they would be source of income for this field. While other specialty teams on occasion make special efforts to support patients and families in these ways, hospice and palliative care stands alone in embracing these categories of human caring. Palliative care clinicians can honestly introduce their services to patients by explaining, “Our palliative care team can provide you and your family with an extra layer of support to improve your comfort and help you live as fully as possible and feel as well as possible throughout your illness.”

This level of caring supports healthy grieving by individuals and families, who might otherwise have had regrets over things left unsaid, forgiveness never asked for or offered, questions that would have gone unasked. In the aggregate, healthy grieving may yield benefits on a scale of population health.

Well-being is the apt term for discussing this aspect of patient experience. Clinicians who care for seriously ill people observe the depth of human suffering, but occasionally—more often when symptoms respond to treatments—also meet people who evince or express feeling well emotionally, socially, or spiritually. Well-being entails emotions of feeling loved and at peace within themselves and with others. Well-being conveys the joy in a person’s face as they hold a grandbaby or the contentment a person feels as they read to a young child or stroke a beloved pet. Sometimes a patient will describe having grown as a person or grown closer to others during their illness.^72^

Within the strategy proposed, this field has an opportunity to rebrand itself as specialists in managing symptoms *and fostering well-being* through the end of life. Conceptually, the field can position its services as the completion of an arc of human caring. This caring continuum begins with family-centered prenatal care, Lamaze classes, La Leche leagues and birth doulas, and extends through hospice and palliative care with guidance and support for the process of completing one’s life drawing on modalities, such as dignity therapy and logotherapy, and services of end-of-life doulas.

An important reason to emphasize *fostering well-being* as a defining feature of hospice and palliative care is the likely impact it would have on public perceptions of the field and its services. Successful brands belong to goods and services that people desire or feel they need. Elevating hospice and palliative care’s brand would help attract more and earlier referrals and increase acceptance by patients and families. The field’s refreshed brand could help hospice and palliative care become a “must have” resource within health systems.

Within the flywheel of competitive health care markets, there are potentially upstream advantages to the field explicitly embracing well-being within its brand. Doing so implicitly redefines high-quality care for patients who are living with a potentially life-limiting illness. Thereafter, for patients and families who must choose between available cancer care centers or heart failure clinics, palliative care can move from an unknown or “nice to have” feature to a “must have” requirement. Primary care providers can make high-quality palliative care and hospice essential criteria for referring their patients.

This is the bright, life-affirming potential that this once-vibrant field can still achieve—and must not retreat from. A renewed commitment to the field’s dual clinical and cultural mission is required. A revitalized field of hospice and palliative care is needed to protect seriously ill patients and their families from harmful health system dysfunctions, prevent and alleviate suffering, and foster well-being throughout their illness.

## Abbreviations

AAHPM: American Academy of Hospice and Palliative Medicine
APP: Advanced Practice Provider
APRN: Advanced Practice Registered Nurse
CAHPS: Consumer Assessment of Healthcare Providers and Systems
CAPC: Center to Advance Palliative Care
CHAP: Community Health Accreditation Partner
CMS: Centers for Medicare and Medicaid Services
CoP: Conditions of Participation (Medicare Hospice Benefit)
DON: Director of Nursing
FTE: Full-Time Equivalent
GIP: General In-Patient
HCI: Hospice Care Index
HHS: U.S. Department of Health and Human Services
HPM: Hospice and Palliative Medicine
ICU: Intensive Care Unit
IDT: Interdisciplinary Team
IPO: Initial Public Offering
MedPAC: Medicare Payment Advisory Commission
MWM: Measuring What Matters
NCI: National Cancer Institute
NHPCO: National Hospice and Palliative Care Organization
NP: Nurse Practitioner
NPHI: National Partnership for Healthcare and Hospice Innovation
OIG: Office of Inspector General
PA: Physician Assistant
PC: Palliative Care
PEF: Private Equity Firm
PET: Positron Emission Tomography
PTC: Publicly Traded Company
TJC: The Joint Commission
VA: Veterans Affairs or Veterans Health Administration
